# Biological Characterization of Hepatitis B virus Genotypes: Their Role in Viral Replication and Antigen Expression

**DOI:** 10.3389/fmicb.2021.758613

**Published:** 2021-11-04

**Authors:** María Mercedes Elizalde, Luciana Tadey, Lilia Mammana, Jorge Fabián Quarleri, Rodolfo Héctor Campos, Diego Martín Flichman

**Affiliations:** ^1^Instituto de Investigaciones Biomédicas en Retrovirus y Sida (INBIRS), CONICET, Universidad de Buenos Aires, Buenos Aires, Argentina; ^2^Consejo Nacional de Investigaciones Científicas y Técnicas (CONICET), Buenos Aires, Argentina; ^3^Unidad de Virología, Hospital de Infecciosas “Francisco J. Muñiz”, Buenos Aires, Argentina; ^4^Departamento de Microbiología, Inmunología, Biotecnología y Genética, Cátedra de Virología, Facultad de Farmacia y Bioquímica, Universidad de Buenos Aires, Buenos Aires, Argentina

**Keywords:** Hepatitis B virus, genotypes, characterization, viral replication, antigen expression

## Abstract

Hepatitis B virus (HBV) inter-host evolution has resulted in genomic diversification reflected in the existence of nine genotypes (A-I) and numerous subgenotypes. There is growing evidence that genotypes influence HBV natural history, clinical outcomes, and treatment response. However, the biological characteristics underlying these differences have not yet been established. By transfecting HuH-7 cells with unit-length constructs of genotypes A2, B2, C1, D1, and F1b, we identified major differences in HBV replicative capacity and antigen expression across genotypes. Genotypes B2 and F1b showed a 2-fold increase in cccDNA levels compared to the other genotypes (*p*<0.005). Genotype A2 expressed the lowest pgRNA levels, with a 70-fold decrease in relation to the other genotypes (*p*<0.0001), while genotype B2 showed the lowest Precore RNA levels, with a 100-fold reduction compared to genotype A2 (*p*<0.0001). The highest intracellular HBV DNA levels were observed for genotype B2 and the lowest for genotypes A2 and C1 (*p*<0.0001). Regarding antigen expression, genotype F1b secreted the highest HBsAg levels and genotype D1 the lowest (*p*<0.0001), while genotypes A2 and B2 showed the highest intracellular HBsAg levels (*p*<0.0001). Interestingly, genotype C1 secreted the highest HBeAg levels, while genotype A2 showed the highest intracellular levels (p<0.0001). Finally, the analysis of the intra/extracellular antigen ratios revealed that most genotypes retained intracellularly 5–20% of the antigens, except the genotype A2 that retained 50% of the total expressed antigens. In conclusion, this study provides new insights into the biological characteristics of HBV genotypes, being the first study to comparatively analyze European (A and D) and Asian (B and C) genotypes with the Latin American (F) genotype. The differences in HBV replication and antigen expression might contribute to understand the differential role of genotypes in pathogenesis.

## Introduction

Hepatitis B virus (HBV) is a major human health problem worldwide, with approximately 296 million individuals chronically infected worldwide and up to 1 million people dying annually from HBV-related liver diseases, such as cirrhosis and hepatocellular carcinoma (HCC) (WHO Hepatitis B, 2017).

HBV is a small, enveloped DNA virus that replicates through an RNA intermediate. The HBV genome consists of a partially double-stranded relaxed circular DNA (rcDNA) of 3.2kb. After infection, host DNA enzymes repaired the rcDNA to generate a covalently closed circular DNA (cccDNA). The cccDNA serves as a transcriptional template for the different viral RNAs. The 3.5kb pregenomic RNA (pgRNA) is the mRNA for the synthesis of polymerase and core proteins and the template for reverse transcription. The 3.5kb Precore mRNA encodes the HBV e antigen (HBeAg). The 2.4kb and 2.1kb transcripts generate the large (LHBs), and medium (MHBs), and small (SHBs) envelope proteins (HBsAg), respectively, while the 0.7kb transcript encodes the regulatory X protein (HBx). In the cytoplasm, core protein subunits assemble pgRNA and viral polymerase to form immature core particles. Once encapsidated, pgRNA is retrotranscribed by the polymerase, and mature rcDNA-containing virions are enveloped and released from the infected hepatocytes (Tsukuda and Watashi, 2020). Alternatively, the rcDNA-containing capsids can re-enter the nucleus through a recycling pathway to replenish the pool of cccDNA. In addition to virions, infected hepatocytes also released filamentous and spherical subviral particles composed primarily of HBsAg proteins (Caballero et al., 2018).

Based on its genetic divergence, HBV has been classified into 9 genotypes (A to I) and numerous subgenotypes. The genotypes (gt) and subgenotypes show different geographical distribution in populations around the globe (Kramvis, 2014; Tong and Revill, 2016). The most cosmopolitan genotypes are A and D, predominant in Europe, Africa, and North America. Genotypes B and C are most frequent in East and South-East Asia, and genotype E is mostly restricted to Africa. Genotype F is autochthonous from the American continent and found in native populations from Alaska, Central, and South America (Shi et al., 2013; Ledesma et al., 2015; Mojsiejczuk et al., 2020).

There is growing evidence that HBV genotypes influence clinical outcomes, HBeAg seroconversion rates, severity of liver disease, emergence of mutants, transmission patterns, and response to interferon therapy (Kim et al., 2011; Liu and Kao, 2013). It has been shown that patients infected with genotype A or B generally respond better to interferon treatment than patients infected with genotypes C and D. In addition, individuals infected with (sub)genotypes C and F1b showed a delayed HbeAg to anti-Hbe seroconversion than those infected with (sub)genotypes A, B, D, and F4. Moreover, growing evidence has shown a close association of (sub)genotypes C and F1b with an early and rapid progression of chronic infection and evolution to HCC (Ching et al., 2016; Marchio et al., 2018; Pineau et al., 2018). However, there is a paucity of data regarding their distinctive biological characteristics, in particular for genotype F, due mainly to the limited geographic distributions of the genotypes.

The majority of studies comparing HBV genotypes have been restricted between genotypes B and C in Asia and genotypes A and D in Europe. In addition, there have been very few *in vitro* studies directly comparing virological parameters across genotypes due to a lack of appropriate replication models (Wose Kinge et al., 2020). Most studies have used more than unit-length HBV constructs. However, in these constructs, the complete HBx open reading frame and the enhancer I/II regions are duplicated. In addition, more than unit-length HBV constructs are directly transcribed in the cell nucleus bypassing the formation of cccDNA replicative intermediate (Amir et al., 2019). Hence, the utilization of these constructs may not be ideal to study differences in HBV replication and protein expression among genotypes. The use of unit-length monomeric constructs without heterologous promoters represents a better alternative due to the absence of duplicated genome regions and the ability of cccDNA formation.

In the present study, by transfecting HuH-7 cells with unit-length monomeric constructs of the most prevalent genotypes in Asia, Europe, and Latin America, we comparatively analyze viral RNA transcription, genome replication, and protein expression across genotypes.

## Materials and Methods

### Viral Constructs

Vector pUC19 containing full-length genomes of HBV genotypes A2, B2, C1, D1, and F1b were constructed for this study. Briefly, HBV DNA was extracted from serum samples of HBeAg-positive chronically infected patients. The characteristics of the patients from whom the serum samples were obtained are shown in Supplementary Table 1. Full-length HBV genomes were amplified adapting the method described by Günther, including *Nde*I/*Bsp*QI sites in P1 sense primer: 5′-CCGGACATATGATGCTCTTCTTTTTCACCTCTGCCTAA TCATC-3′, and *Sac*I/*Bsp*QI sites in P2 antisense primer (5′-CCGGAGAGCTCATGCTCTTCAAAAA GTTGCATGGTG CTGGTG-3′). Polymerase chain reactions (PCR) were performed using the Expand high-fidelity PCR system (Roche, Mannheim, Germany) according to the manufacturer’s instructions.

The amplified HBV DNAs were digested with *Nde*I and *Sac*I restriction enzymes (New England Biolabs, Beverly, MA, United States). The 3.2kb fragments were separated by agarose gel electrophoresis, recovered by gel purification using the PureLink Quick Gel Extraction Kit (Invitrogen, Carlsbad, CA, United States), and inserted into *Nde*I/*Sac*I sites of pUC19 vector. After cloning, constructs were sequenced in order to confirm the absence of mutations that might influence HBV replication and/or protein expression.

The subgenotypes used in the present study were selected according to the following criteria: the most widely distributed of their respective genotypes and the best characterized in previous studies (Velkov et al., 2018; Araujo et al., 2020; McNaughton et al., 2020).

Subsequently, among the 242 positive samples for HBsAg and HBeAg (A2: 53, B2: 16, C1: 17, D1: 48 and F1b: 108) available in our sample bank, those closest phylogenetically to the consensus of each subgenotype obtained from the HBVdb database^1^ were cloned. Sequences containing deletions, mutations associated with HBeAg loss, or antiviral treatment were excluded from the analysis.

The GenBank accession numbers for these constructs were as follows: OK106253 (genotype A2), OK106254 (genotype B2), OK106255 (genotype C1), OK106256 (genotype D1), and OK106257 (Genotype F1b).

### Cell Culture and Transfection

Human hepatoma cell lines HuH-7 (JCRB Cell Bank #0403) and HepG2 (ATCC HB-8065) were grown in Dulbecco’s modified Eagle’s medium (DMEM; Sigma, CA, United States) supplemented with 10% fetal bovine serum (Sigma, CA, United States), 1mm nonessential amino acids (GIBCO, Carlsbad, CA, United States), 2mmL-glutamine (GIBCO, Carlsbad, CA, United States), 0.15% sodium bicarbonate, 100 UI/ml penicillin, and 100μg/ml streptomycin at 37°C with 5% CO_2_.

Full-length linear HBV DNAs (nt 1820–1820) with sticky ends were used for transfections. As previously described, linear HBV monomers were excised from the plasmids by restriction enzyme digestion with 5U of *Bsp*QI (New England Biolabs, Beverly, MA, United States) at 50°C. The 3.2kb fragments were gel purified with PureLink Quick Gel Extraction Kit (Invitrogen, Carlsbad, CA, United States), according to the manufacturer’s instructions, and the DNA was quantified by spectrometry (Elizalde et al., 2019). In order to mimic viral variability, a mix of 10 to 20 clones (pUC19-full-length HBV genomes) of each genotype were used in all experiments.

Cells were seeded to semi confluence in 6- or 24-well plates and transfected with 1μg of full-length HBV DNAs for 6-well plates and 0,25 μg for the 24-well plates. Transient transfections were carried out using X-tremeGene^™^ 9 transfection reagent (Roche, Mannheim, Germany), according to the manufacturer’s recommendations. The cells were maintained at 37°C in 5% CO_2_ atmosphere. After 6h, the medium was replaced, and the cultures were incubated for 72h. In experiments to evaluate cccDNA recycling, cells were treated with 100μm Lamivudine throughout the assay.

In all experiments, 0.05μg of the luciferase reporter vector pGL4.13 (luc2/SV40; Promega, Madison, WI, United States) was co-transfected with the linear full-length HBV genomes as transfection efficiency control. To evaluate the light emission produced by the luciferase activity, 20μl of cell lysates was mixed with the commercial reagent Luciferase Assay System (Promega, Madison, WI, United States), according to the manufacturer’s recommendations, and relative light units were detected. Results were expressed per relative light units.

### Analysis of Covalently Closed Circular DNA

The quantification of HBV cccDNA was performed by quantitative real-time PCR (qPCR). Briefly, 72h post-transfection, cells were treated with lysis buffer containing 50mm Tris-HCl (pH 8), 10mm EDTA, 100mm NaCl, 0.5% SDS, and 0.5mg/ml proteinase K (Invitrogen, United States) and incubated at 56°C for 2h. Nucleic acids were purified by phenol-chloroform extraction and ethanol precipitation. Isolated intracellular total DNA was treated with 500U/ml of T5 exonuclease (New England Biolabs, Beverly, MA, United States) at 37°C for 1h to remove non-supercoiled dsDNA. Samples were subjected to qPCR using Luna Universal qPCR Master Mix (2x; New England Biolabs, Beverly, MA, United States). Selective cccDNA primers: sense 5’-GTCTGTTCCTTCTCATCTGC-3′ (nt 1551–1570) and antisense 5’-AGGCACAGCTTG GTGGCTTG-3′ (nt 1887–1868) were used for the qPCR. Mitochondrial DNA was analyzed as an internal reference to normalized cccDNA levels. The primers for detection of mitochondrial DNA were as follows: sense 5′-CCCCACAAACCCCATTACTAAACCCA-3′ and antisense 5′-TTTCATCATGCGGAGATGTTGGATGG-3′. Serial dilutions of a HBV replication-competent plasmid (pCH-9/3091) were used as quantification standards. HBV DNA extracted from serum samples of HBeAg-positive patients with a viral load of 8.2 Log10 IU/ml was used as a control to evaluate the efficacy of DNase treatment and the specificity of the cccDNA amplification.

### Analysis of HBV RNA

Precore mRNA and pgRNA were quantified by RT-qPCR. Total cellular RNA from transfected cells was extracted with TRIzol reagent (Invitrogen, Carlsbad, CA, United States). RNA samples were treated with RQ1 RNase-free DNase (Promega, Madison, WI, United States) at 37°C for 1h, to remove DNA. Concentration and purity of RNA were determined by spectrometry. One microgram of RNA was reverse-transcribed into cDNA with Random Hexamer Primers (Biodynamics, Buenos Aires, Argentina) using M-MLV reverse transcriptase (Promega, Madison, WI, United States). Then Precore mRNA and pgRNA were quantified as previously described (Laras et al., 2002, 2006). Briefly, the cDNA product was used in two separate amplification reactions with a common antisense primer: 5′-GGAAAGAAGTCAGAAGGCAA-3′ (nt 1974–1955), and sense primers: 5′-GGTCTGCGCACCAGCACC-3′ (nt 1796–1813) for the specific detection of Precore mRNA transcripts and 5’-CACCTCTGCCTAATCATC-3′ (nt 1826–1843) primer for detecting total Core Promoter directed transcription. The levels of pgRNA transcripts were calculated by subtracting Precore mRNA levels from total Core Promoter directed transcription. Amplification of GAPDH cDNA was used as housekeeping gene to normalize the mRNA levels. Primers used for GAPDH mRNA amplification were as follows: sense 5′-GAAGGTGAAGG TCGGAGTC-3′ and antisense 5’-GAAGA TGGTGATGGG ATTTC-3′. For quantification, serial dilutions of an HBV replication-competent plasmid were used as standards.

To rule out amplification of contaminating DNA, PCR amplification was also routinely performed without reverse transcriptase.

HBV replicative activity (replication fitness) was expressed as pgRNA/cccDNA ratio, while Precore mRNA/cccDNA ratio was used to assess HBV transcriptional efficiency.

### Analysis of Intracellular HBV DNA

HBV replicative intermediates were analyzed by Southern blot. Briefly, 72h post-transfection, cells were treated with lysis buffer (50mm Tris/HCl pH 7.5; 100mm NaCl; 1mm EDTA; 0.5% NP40) and centrifuged 1min at 14,000rpm to remove the nuclei. The supernatants were collected and treated with 0.5mg/ml proteinase K (Invitrogen, Carlsbad, CA, United States) at 56°C for 2h. Nucleic acids were purified by phenol-chloroform extraction and ethanol precipitation in the presence of 20μg of dextran. DNA isolated from cell lysates was separated on a 1% agarose gel and blotted onto a nylon-positive membrane (Roche, Mannheim, Germany). The transferred DNA was immobilized by an ultraviolet crosslinker and hybridized with a subgenomic digoxigenin (DIG)-labelled probe (Roche, Mannheim, Germany) of each genotype. The hybridization signals were detected on an X-ray film using an enzyme-linked immunoassay (DIG Luminescent Detection Kit; Roche, Mannheim, Germany) and were quantified with the ImageJ software (Wayne Rasband, NIH, United States).

### Analysis of Extracellular HBV DNA

Viral progeny was quantified by qPCR. Seventy-two hours post-transfection, cell culture supernatants were harvested and clarified by centrifugation at 3,000g for 10min. Supernatants were treated with lysis buffer (50mm Tris-HCl pH 7.5; 1mm EDTA; 1% SDS; 0.5mg/ml proteinase K (Invitrogen, United States)) and incubated at 56°C for 2h. Nucleic acids were purified by phenol-chloroform extraction and ethanol precipitation in the presence of 20μg of dextran. qPCR was performed in a Step One Plus Real-Time PCR System (Applied Biosystems, Foster City, CA, United States) using Luna Universal qPCR Master Mix (2x; New England Biolabs, Beverly, MA, United States). The following primers were used for the amplification: sense 5’-ATGGAGACCACCGTGAACGC-3′ (nt 1608–1627) and antisense 5′-AGGCACAGCTTG GTGGCTTG-3′ (nt 1887–1868). To avoid amplification of the input linear HBV DNA used for transfection, primers that specifically amplify relaxed circular HBV DNA were used (Elizalde et al., 2019). Serial dilutions of an HBV replication-competent plasmid (pCH-9/3091) were used as quantification standards.

### Antigen Quantification

The HBsAg concentration in cell lysates and cell culture supernatants was measured by electrochemiluminescence immunoassay (ECLIA) using the Elecsys HBsAg II quant II on a Cobas e801 instrument (Roche, Mannheim, Germany), according to the instructions of the manufacturer. Results were expressed in IU/ml. The HBeAg concentration in cell lysates and culture supernatants was measured using the Elecsys HBeAg on a Cobas e801 instrument (Roche, Mannheim, Germany), according to the manufacturer’s recommendations. Results were expressed in Sample/Cut off value (S/CO).

The linearity dynamic range of the assay was validated making serial dilutions of serum samples with known HBeAg levels.

To rule out the possibility of cross-reactivity of the core protein and HBeAg, HuH-7 cells were transfected with a full-length HBV genome harboring the G1896A Precore mutation. Furthermore, the possible interference of the intracellular cell lysate in the specificity of the assay was evaluated by challenging serum samples with known HBeAg or qHBsAg levels diluted with non-transfected cell lysates.

### Western Blot Analysis

For intracellular HBV surface proteins, cells were lysed in 1% Triton, 20mm Tris, 1mm EDTA, 150mm NaCl, and protease inhibitor cocktail (Sigma, United States). For extracellular proteins, cell culture supernatants were used directly. Samples were mixed in Laemmli buffer, boiled, loaded on 12% SDS-polyacrylamide gels, and transferred to PVDF membranes (Hybond, GE Healthcare, UK), according to standard protocols. The membranes were blocked in 5% non-fat milk in Tris-buffered saline (20mm Tris and 150mm NaCl, pH 7.6) containing 0.1% Tween-20 for 1h at room temperature. HBV surface proteins were detected using anti-SHBs (HB01) monoclonal antibody (kindly provided by Prof. Aurelia Zvirbliene, Lithuania). Peroxidase-conjugated secondary antibodies were purchased from Santa Cruz Biotechnology (United States). Protein-specific bands were visualized using an enhanced chemiluminescence (ECL) system (GE Healthcare, UK) by autoradiography. The quantification was performed using ImageJ analysis software (Wayne Rasband, NIH, United States). β-actin detection was used as intracellular protein loading control.

### Statistical Analysis

All experiments were performed independently three times. Statistical significance was determined by one-way ANOVA followed by *post hoc* Turkey’s test. A value of *p*<0.005 was considered statistically significant. Results were expressed as mean±standard deviation. All analyses were performed using GraphPad Prism 8 software (GraphPad Software, San Diego, CA, United States).

## Results

### HBV Replicative Capacity Varied Distinctly Across Genotypes

To comparatively analyze the replication capacity among HBV genotypes, three days post-transfection, cells and culture supernatants were harvested, and levels of cccDNA, viral RNA, intracellular HBV DNA, and secreted virion DNA were examined.

#### Covalently Closed Circular DNA

**Figure 1:**
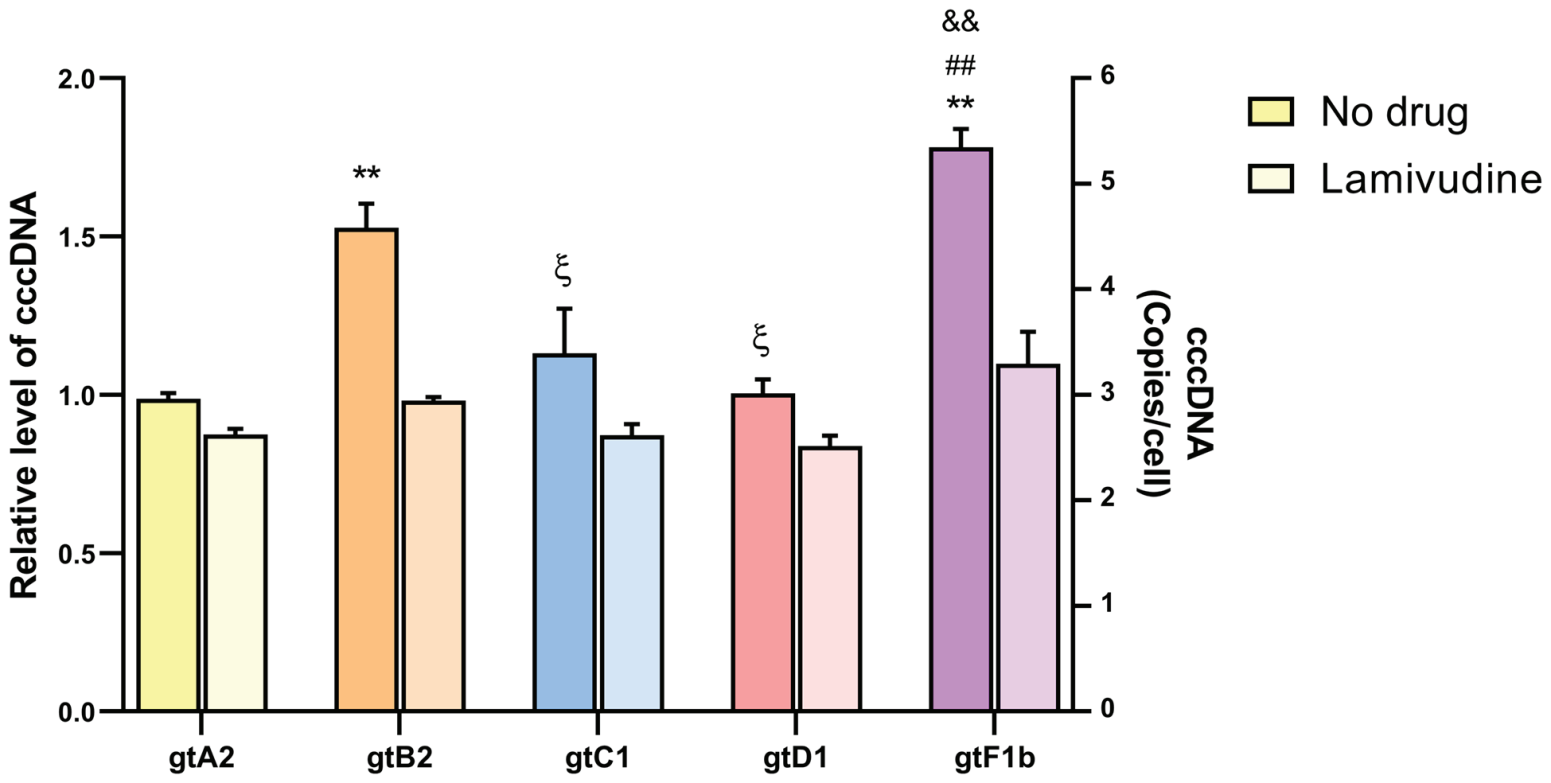
Analysis of cccDNA levels among genotypes. HuH-7 cells were transfected with linear full-length HBV genomes of genotypes A2, B2, C1, D1, and F1b in the presence or in the absence of 100μm Lamivudine. 3days post-transfection, cells were harvested, total DNA was extracted, and cccDNA levels were assessed by qPCR. Results were normalized to mitochondrial DNA. Shown values represent the mean±standard deviation of three independent experiments. ^*^: statistical difference in relation to gtA2, ξ: difference in relation to gtB2, #: difference in relation to gtC1 and &: difference in relation to gtD1. One symbol: *p*<0.005 and two symbols: *p*<0.0001.

Significant differences were observed in cccDNA levels among genotypes. As shown in Figure 1, genotypes B2 and F1b showed an approximately 2-fold increase compared to genotypes A2, C1, and D1 (*p*<0.005, in comparison with genotype A2). In all cases, the values were normalized to those of genotype A2.

In order to assess whether cccDNA recycling was involved in the observed differences in cccDNA levels among genotypes, transfection assays were performed in the presence of 100μm Lamivudine to inhibit reverse transcription, formation of new rcDNA, and replenish of cccDNA pool (Kock et al., 2003). Lamivudine treatment reduced extracellular HBV DNA levels by more than 99% (Supplementary Figure 1). Interestingly, cccDNA levels were similar among genotypes in the presence of Lamivudine, in contrast to the differences observed in the absence of the antiviral (Figure 1). This result suggests that the differences in cccDNA levels across genotypes might be related to an unequal recycling capacity among HBV genotypes.

#### HBV RNA

**Figure 2:**
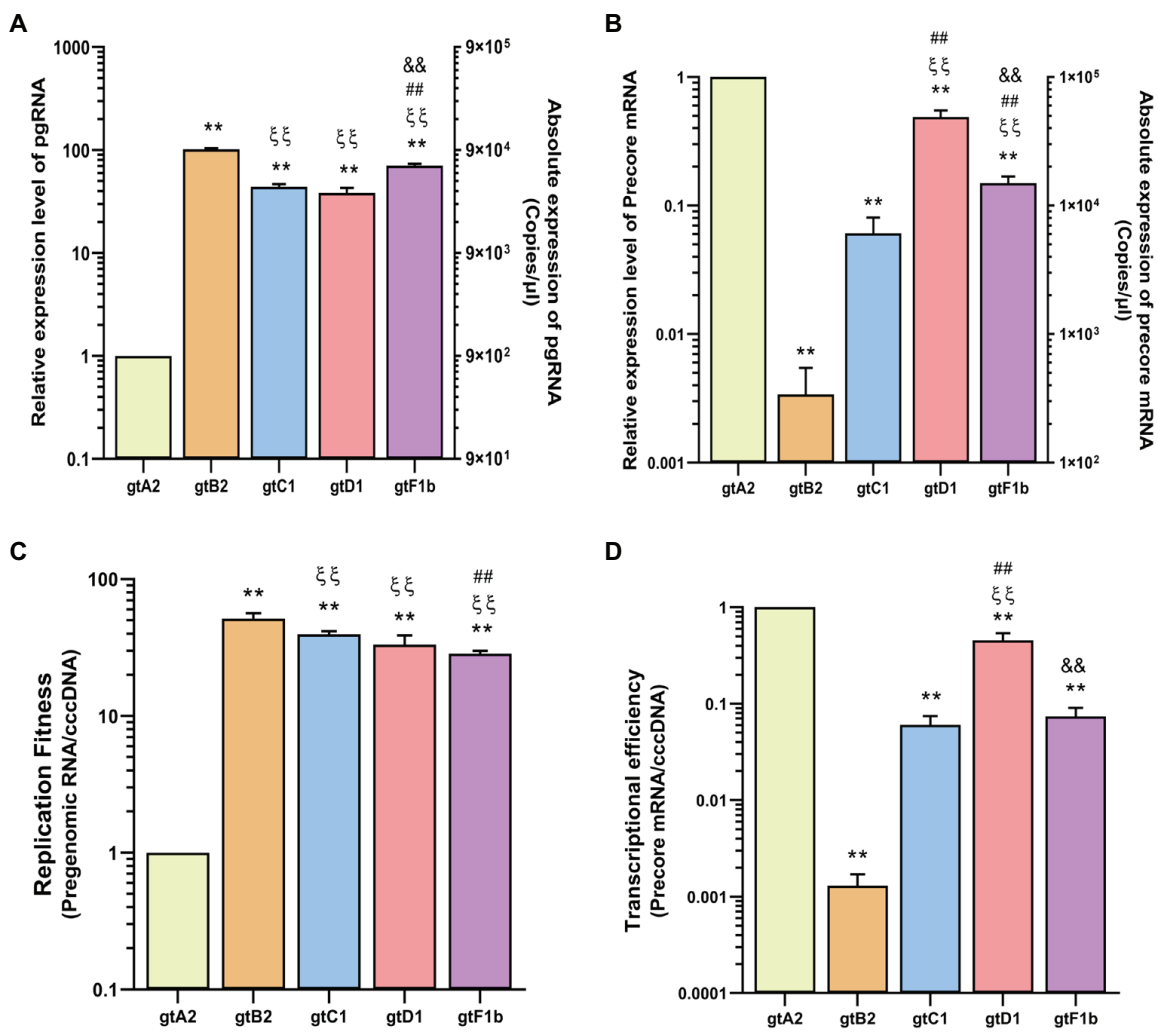
Analysis of HBV RNA levels across genotypes. HuH-7 cells were transfected with linear full-length HBV genomes of genotypes A2, B2, C1, D1, and F1b. Three days post-transfection, cells were harvested, total RNA was extracted, and pgRNA **(A)** and Precore mRNA **(B)** levels were analyzed by transcript-specific RT-qPCR. **(C)** cccDNA replicative activity was determined as molecules of pgRNA synthesized per cccDNA (pgRNA/cccDNA). **(D)** The number of Precore mRNA transcripts produced per cccDNA (Precore RNA/HBVccc) was used to assess HBV transcriptional efficiency. Values shown represent the mean±standard deviation of three independent experiments. ^*^: difference in relation to gtA2, ξ: difference in relation to gtB2, #: difference in relation to gtC1 and &: difference in relation to gtD1. Two symbols: p<0.0001.

The pgRNA levels also revealed notable differences among genotypes (Figure 2A). Genotype A2 expressed the lowest levels of pgRNA transcripts, while genotypes C1, D1, and particularly genotypes B2 and F1b expressed the highest levels of pgRNA, with an increase of more than 70 times in relation to genotype A2 (*p*<0.0001, in comparison with genotype A2). Whereas Precore mRNA levels showed that genotypes A2 and D1 expressed the highest levels, while genotypes B2 and C1 presented the lowest levels, with a reduction of more than 100-fold compared to genotype A2 (*p*<0.0001, in comparison with genotype A2; Figure 2B).

These results showed a notable imbalance in the transcription of the RNAs regulated by the Core Promoter. In some genotypes (B2, C1, and F1b), pgRNA levels exceed Precore mRNA by more than two orders of magnitude, while in other genotypes (A2 and D1), the pgRNA/Precore mRNA ratio was significantly lower.

In addition, the pgRNA/cccDNA ratio revealed that pgRNA levels reflected the replicative activity of the cccDNA, which is a very low replicative activity for genotype A2, compared to the other genotypes (*p*<0.0001, in comparison with genotype A2; Figure 2C). On the other hand, regarding the Precore mRNA/cccDNA ratio, genotypes B2 and C1 showed the lowest transcriptional activity of the cccDNA, while genotypes A2 and D1 presented the highest transcriptional activity (*p*<0.0001, in comparison with genotype A2; Figure 2D).

#### HBV DNA

**Figure 3:**
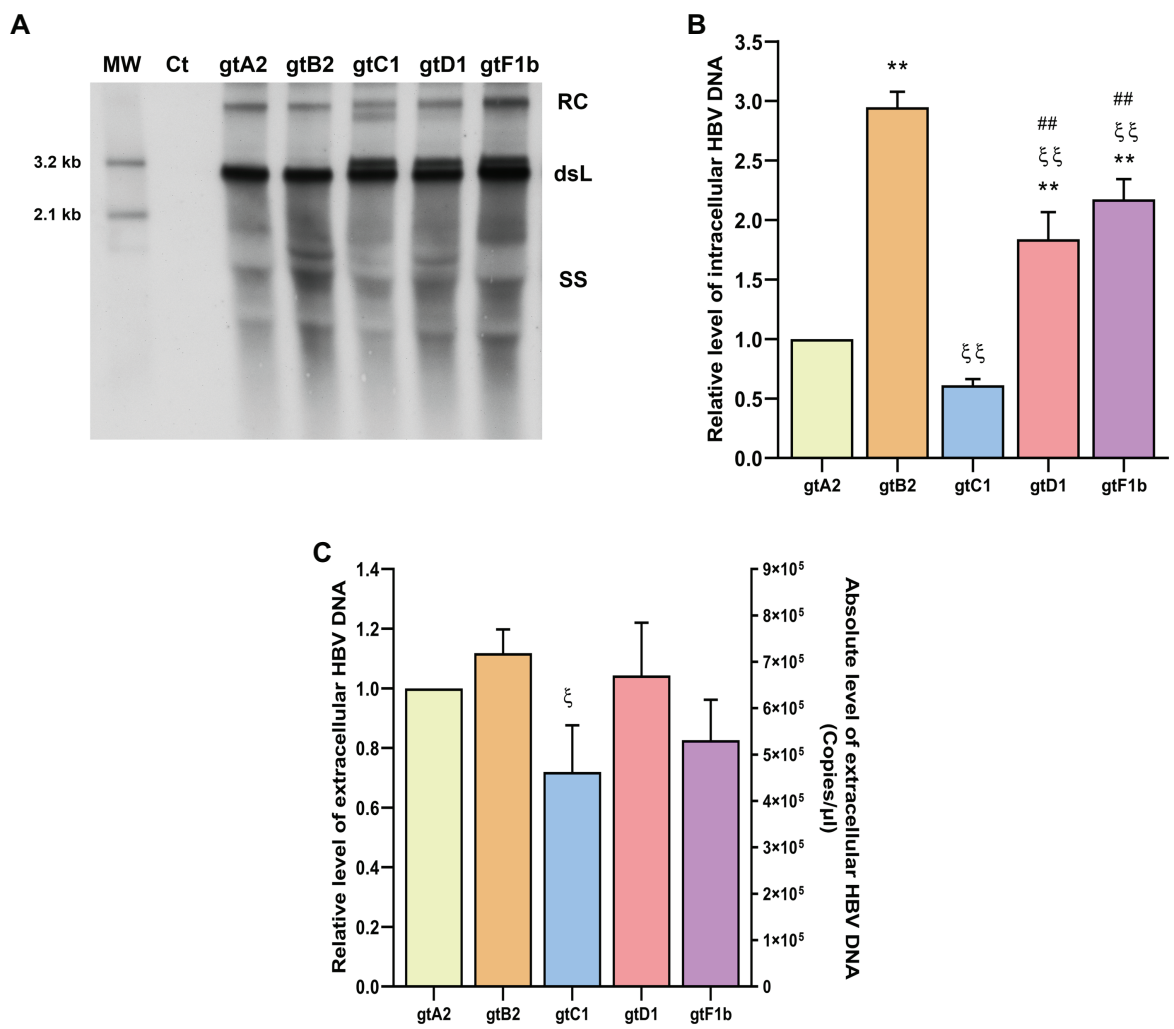
Analysis of HBV DNA levels among genotypes. HuH-7 cells were transfected with linear full-length HBV genomes of genotypes A2, B2, C1, D1, and F1b. Three days post-transfection, cells and culture supernatants were harvested. **(A)** HBV DNA replicative intermediates were assessed by Southern blot analysis. Ct: cell transfected with pUC19 empty vector (control); RC: HBV relaxed circular DNA; dsL: HBV double-stranded linear DNA; SS: HBV single-stranded DNA. **(B)** Relative intensity of the SS band was quantified using ImageJ software. The band corresponding to the dsL HBV DNA was not included in the quantitative analysis because this DNA may be partially derived from transfected input DNA. **(C)** HBV extracellular DNA levels were determined by qPCR. Shown values represent the mean±standard deviation of three independent experiments. ^*^: difference in relation to gtA2, ξ: difference in relation to gtB2, #: difference in relation to gtC1 and &: difference in relation to gtD1. One symbol: *p*<0.005 and two symbols: *p*<0.0001.

The analysis of intracellular HBV DNA intermediates also revealed marked differences between genotypes (Figures 3A,B). The highest levels were observed for genotype B2 (*p*<0.0001, in comparison with genotype A2), reflecting similar findings for cccDNA and pgRNA. In contrast, the lowest DNA levels were detected for genotypes A2 and C1 (*p*<0.0001, in comparison with genotype A2). For genotype A2, this result is in line with cccDNA and pgRNA levels. Whereas for genotype C1, intracellular DNA levels correspond to those of cccDNA, but did not mirror pgRNA levels.

Interestingly, the notable differences in the levels of cccDNA, pgRNA, and intracellular HBV DNA were not strictly reflected in the extracellular HBV DNA levels. No significant differences were observed in the extracellular HBV DNA levels in relation to genotype A2 (Figure 3C). Additionally, similar findings were also obtained when these clones were transfected in HepG2 cells (Supplementary Figure 2).

### HBV Protein Expression and Secretion Differ Among Genotypes

To comparatively analyze the expression and secretion of HBV proteins across genotypes, cells and culture supernatants were harvested from transfected cells and the intra- and extracellular levels of HBsAg and HBeAg were determined.

#### HBsAg

**Figure 4:**
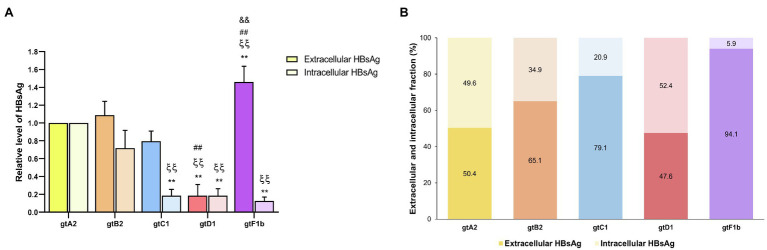
Analysis of intracellular and secreted HBsAg levels across genotypes. HuH-7 cells were transfected with linear full-length HBV genomes of genotypes A2, B2, C1, D1, and F1b. Three days post-transfection, cells and culture supernatants were harvested, and intracellular and extracellular levels of HBsAg were determined by electrochemiluminescence immunoassay **(A)**. Extracellular/intracellular HbsAg ratio **(B)**. Values shown represent the mean±standard deviation of three independent experiments. ^*^: Intracellular or extracellular difference in relation to genotype A2, ξ: Intracellular or extracellular difference in relation to genotype B2, #: Intracellular or extracellular difference in relation to genotype gtC1 and &: Intracellular or extracellular difference in relation to genotype D1. Two symbols: p<0.0001.

At the extracellular level, genotype F1b secreted the highest HBsAg levels, whereas genotype D1 secreted significantly lower levels (*p*<0.0001, in comparison with genotype A2). In contrast, the analysis of intracellular HBsAg revealed that genotypes A2 and B2 expressed the highest levels, while genotypes C1, D1, and F1b showed the lowest levels (*p*<0.0001, in comparison with genotype A2; Figure 4A). Regardless of the absolute amount of HBsAg, it is worth noting the differences in the ratio of HBsAg expression and secretion across genotypes. While genotype F1b secreted more than 90% of the antigen, genotypes A2 and D1 retained approximately 50% of the total protein in the cells (Figure 4B). Similar patterns of HBsAg expression and secretion across genotypes were also observed after transfection in HepG2 cells (Supplementary Figure 3). As in the evaluation of viral replication, the mean value of genotype A2 was used to normalize the samples.

**Figure 5:**
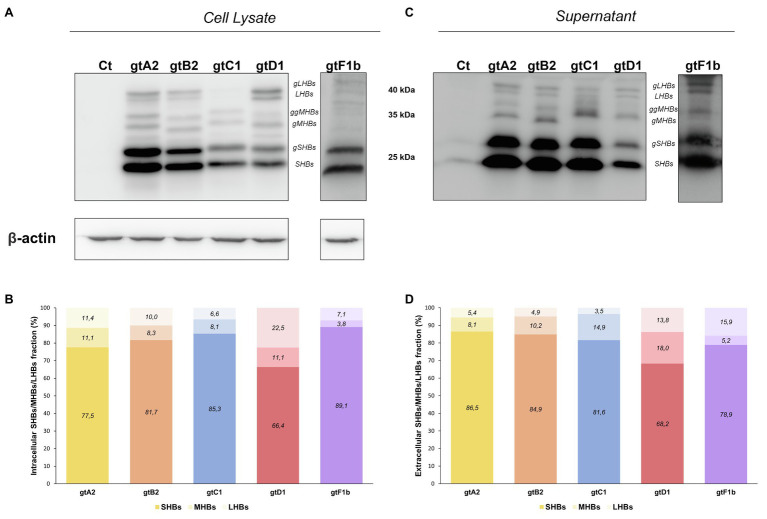
Relative composition of HBsAg proteins among genotypes. HuH-7 cells were transfected with linear full-length HBV genomes of genotypes A2, B2, C1, D1, and F1b. Three days post-transfection, cellular lysates **(A)** and the corresponding culture supernatants **(B)** were analyzed by Western Blot, using a SHBs specific monoclonal (HB01) antibody. As this antibody shows a decreased reactivity for HBsAg derived from genotype F transfected cells, an increased time of exposure was needed. SHBs and LHBs occur in unglycosylated (LHBs and SHBs) and glycosylated (gLHBs and gSHBs) forms, while MHBs occurs in glycosylated (gMHBs) and double glycosylated (ggMHBs) forms **(C)** Intracellular and **(D)** extracellular LHBs/MHBs/SHBs ratio was determined. Ct: cell transfected with pUC19 empty vector (control).

In order to further characterize HBsAg expression among genotypes, the relative abundance of the different surface proteins (LHBs, MHBs, and SHBs) was determined. For all genotypes, it was observed that SHBs (24 and 27kDa) represented the major fraction of HBsAg, both intra- and extracellularly (Figure 5). However, the relative amount of LHBs/MHBs compared to SHBs was higher for genotype D1 (> 30%) in comparison with the rest of the genotypes (< 23%). Regarding the relative amount of LHBs (42 and 39kDa) and MHBs (36 and 33kDa), no significant differences were observed across genotypes in cell lysates; however, in the supernatants, a greater amount of MHBs than LHBs was observed for genotypes A2, B2, C1, and D1, while for genotype F1b, this ratio was inverted.

#### HBeAg

**Figure 6:**
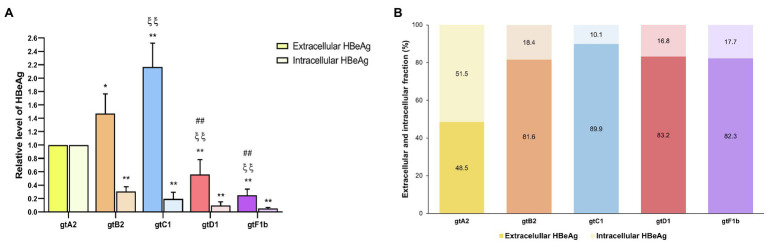
Analysis of intracellular and secreted HBeAg levels across genotypes. HuH-7 cells were transfected with linear full-length HBV genomes of genotypes A2, B2, C1, D1, and F1b. Three days post-transfection, cells and culture supernatants were harvested, and intra- and extracellular levels of HBeAg were determined by ECLIA **(A)**. Extracellular/intracellular HBeAg ratio **(B)**. Shown values represent the mean±standard deviation of three independent experiments. ^*^: Intracellular or extracellular difference in relation to genotype A2, ξ: Intracellular or extracellular difference in relation to genotype B2, and #: Intracellular or extracellular difference in relation to genotype gtC1. One symbol: p<0.005 and two symbols: *p*<0.0001.

The extracellular HBeAg analysis showed that genotype C1, one of the lowest replicators, secreted the highest levels, followed by genotypes B2 and A2, while genotypes D1 and F1b secreted the lowest levels of HBeAg (*p*<0.0001, in comparison with genotype A2). On the contrary, the intracellular HBeAg analysis revealed that genotype A2 expressed significantly higher levels in relation to the rest of the genotypes (*p*<0.0001, in comparison with genotype A2; Figure 6A). Furthermore, the evaluation of the intra/extracellular HBeAg ratio showed that most genotypes retained 10–20% of the antigen in the cells, except for genotype A2 which retained 50% of the protein intracellularly (Figure 6B). In a similar way, comparable levels of intra- and extracellular HBeAg were detected in HepG2 cells (Supplementary Figure 4).

To rule out the possibility of cross-reactivity of the core protein and HBeAg, HuH-7 cells were also transfected with a full-length HBV genome harboring the G1896A Precore mutation that abrogates HBeAg expression. Intra- and extracellular HBeAg was not detected in the Precore mutant (Supplementary Figure 5), indicating that there is no cross-reactivity between HBV core protein and HBeAg in the ECLIA assay.

Taken together, these results showed that HBV genotypes differ in their replication capacity, as well as in their protein expression and secretion. As a summary, the overall expression of each genotype is shown in Table 1.

**Table 1:** Viral replication and protein expression levels of each genotype.

	Genotype				
	A2	B2	C1	D1	F1b
cccDNA (Copies/cell)	2.73	4.71^**^	3.34^ξ^	3.13^ξ^	5.34^** ## &&^
pgRNA (Copies/μl)	9.31×10^2^	9.32×10^4** ξξ^	4.1×10^4** ξξ^	3.58×10^4** ξξ^	6.58×10^4** ξξ ## &&^
pgRNA (Copies/μl)	1.02×10^5^	3.48×10^2**^	6.25×10^3**^	5.02×10^4** ξξ ##^	1.54×10^4** ξξ ## &&^
Intracellular DNA (OD)	3.89×10^3^	1.15×10^4**^	2.38×10^3ξξ^	7.16×10^3** ξξ ##^	8.44×10^3** ξξ ##^
Extracellular DNA (Copies/μl)	6.59×10^5^	7.38×10^5^	4.74x10^5ξ^	6.59×10^5^	5.47×10^5^
Extracellular HBsAg (IU/ml)	99.7	108.67	79.76	18.94^** ξξ ##^	145.56^** ξξ ## &&^
Intracellular HBsAg (IU/ml)	95.9	69.04	18.22^** ξξ^	18.21^** ξξ^	12.47^** ξξ^
Extracellular HBeAg (S/Co)	26.3	38.66^*^	57.07^** ξξ^	14.73^** ξξ ##^	6.58^** ξξ** ξξ ##^
Intracellular HBeAg (S/Co)	27.9	8.65^**^	5.52^**^	2.76^**^	1.47^**^

*Shown values represent the mean of three independent experiments*. ^*^, difference in relation to gtA2; ^ξ^, difference in relation to gtB2; ^#^, difference in relation to gtC1 and ^&^, difference in relation to gtD1. One symbol: p<0.005 and two symbols: p<0.0001.

## Discussion

The HBV replication hinges on a finely poised and complex interplay between several viral factors, including viral genotypes. There is growing evidence that HBV genotypes differ in clinical outcomes, response to antiviral treatments, progression of liver disease, among other features (Kim et al., 2011; Liu and Kao, 2013); however, the molecular basis or biological properties of the virus that support these differences have not yet been established.

High viral load levels and the HBeAg-positive stage have been identified as markers associated with the risk of liver disease progression. Likewise, the intracellular accumulation of viral proteins has been described as a direct mechanism of pathogenesis in chronic HBV infection (Seeger and Mason, 2015; Hadziyannis and Laras, 2018).

In the present study, we characterize the biological properties of HBV genotypes A2, B2, C1, D1, and F1b, identifying major differences in HBV replicative capacity and antigen expression across these genotypes.

Even though previous works have functionally characterized HBV genotypes (Sugiyama et al., 2006; Qin et al., 2011; Sozzi et al., 2016, 2018; Bannister et al., 2020), this is the first study to comparatively analyze the European (A and D) and Asian (B and C) genotypes with the indigenous American F genotype. Genotype F shows a wide distribution throughout the American continent and is the autochthonous strain in native communities from Central and South America, being also found in Alaska (Ledesma et al., 2015; Rodrigo et al., 2016; Mojsiejczuk et al., 2020). Therefore, the functional characterization of American (sub)genotypes and their comparison to genotypes circulating in other geographical regions would provide valuable information to try to understand the differences in HBV natural history observed across HBV (sub)genotypes.

In this study, we have used unit-length monomeric HBV genomes, which represent a more reliable transfection system to study HBV replicative capacity across genotypes. Previous studies comparing the biological properties of genotypes have used more than unit-length constructs, which may influence the replication capacity and antigen expression of HBV genotypes (Bhoola et al., 2014; Wose Kinge et al., 2020). Even though this is a more robust system, promoting high levels of HBV replication and protein expression, the enhancer I/II and the Core promoter regions that regulate the transcription of pgRNA and Precore mRNA, are duplicated. In addition, more than unit-length HBV constructs are directly transcribed in the cell nucleus regardless of the formation of cccDNA (Amir et al., 2019). Therefore, these features may alter the biological properties of the constructs, which is unsuitable for evaluating differences in HBV replication among genotypes.

In the present study, we showed significant differences in HBV replication capacity among genotypes, being higher in genotype B2, followed by genotypes F1b and D1, whereas genotypes A2 and C1 showed the lowest replication capacity. These findings are consistent with those of Qin et al., who identified a higher replication capacity for genotype B2 in relation to genotype C after transfecting HuH-7 cells with circularized HBV genomes (Qin et al., 2011). In contrast, other studies have shown higher replication rates for genotypes A2, C2, and D3 compared to genotype B2 (Sozzi et al., 2016) or higher rates for genotype C compared to genotypes B2 and A2 (Sugiyama et al., 2006) following transfection of HuH-7 cells with 1.3- or 1.24-fold copy of the HBV genome, respectively. These controversial results might be a consequence of the different experimental systems used in the studies, as well as the subgenotypes utilized, since dissimilar biological characteristics have been described even between subgenotypes of the same genotype (Bhoola and Kramvis, 2016; Khatun et al., 2018; Elizalde et al., 2019).

The uneven replicative capacity among genotypes observed in this work might contribute to explain the clinical differences observed across HBV (sub)genotypes. On the one hand, genotypes A and C have been associated with a higher progression to chronic infection in relation to genotypes D and B, respectively (Sumi et al., 2003; Chu and Liaw, 2005; Liu and Kao, 2013). The low replicative activity of these genotypes might contribute to the immune response evasion, favoring the persistence of the infection. On the other hand, genotype B has been associated with fulminant hepatitis and acute liver failure (Ozasa et al., 2006; Ren, 2010). The intracellular accumulation of replicative intermediates in genotype B infected patients might provoke a more vigorous though short-lived immune clearance phase which could trigger fulminant hepatitis and liver failure. However, extrapolation of results from *in vitro* experimental models directly to the clinic should be done with caution.

The Core promoter plays a key role in HBV replication and morphogenesis, directing the transcription of both pgRNA and Precore mRNA (Quarleri, 2014). The specific detection and quantification of these transcripts revealed striking differences among genotypes. In genotypes A2 and D1, the Core promoter activity is diverted to the production of Precore mRNA, whereas in genotypes B2, C1, and F1b, the activity of the Core promoter is biased to the production of pgRNA. *In vitro* analysis of mutants of this region has established that the Core promoter contains cis-acting elements that independently direct the transcription of pgRNA and Precore mRNA. These two elements overlap slightly yet are genetically distinct (Chen et al., 1995; Günther et al., 1997). In addition, it has been shown that several transcriptional factors bind to these regulatory elements and differentially regulate the synthesis of pgRNA and Precore mRNA (Yu and Mertz, 1997; Zheng et al., 2004; Meier-Stephenson et al., 2018). Sequence analysis of the Core promoter region revealed high levels of sequence variability across genotypes (range from 6 to 15% of divergence). The imbalance in the transcription of the RNAs regulated by the Core promoter may be a consequence of the sequence variability across genotypes, causing a differential binding of transcription factors in these two regulatory regions.

Paradoxically, while lower levels of cccDNA, pgRNA, and intracellular HBV DNA were detected for genotypes A2 and C1 in relation to genotypes B2 and F1b, similar levels of extracellular DNA were observed in all genotypes. These findings are in line with previous studies that showed a lower replication level, but a higher virion secretion capacity for genotype C in relation to genotype B (Qin et al., 2011), suggesting that the low replication capacity combined with the efficient virion secretion for genotype C would lead to the rapid spread of the virus without eliciting a strong immune response, contributing to persistent infections.

Furthermore, after pgRNA is encapsidated and retrotranscribed into rcDNA form, immature capsids can be enveloped and released from the infected hepatocytes as virions or, alternatively, recycled back into the nucleus, to maintain the reservoir of cccDNA. Our results suggest that genotypes B2 and F1b might favor the recycling path, increasing the cccDNA pool over virion secretion; meanwhile, genotypes A2 and C1 would favor the secretion of the viral particles. This supports the comparable levels of extracellular HBV DNA despite the observed differences in cccDNA levels among genotypes.

The analysis of antigen expression and secretion also revealed remarkable differences among genotypes. Overall, the different genotypes expressed comparable levels of total HBsAg, except for genotype D1, which synthesized substantial lower levels of the antigen. Similar findings were also described in several *in vitro* studies (Sugiyama et al., 2006; Sozzi et al., 2016; Hassemer et al., 2017; Zhang et al., 2017), as well as in the serum of patients infected with different HBV genotypes (Brunetto et al., 2013; Riveiro-Barciela et al., 2017; Salpini et al., 2020). HBsAg expression is regulated by two distinct promoters: Surface promoter I (SPI) regulates the transcription of the 2.4-kb mRNA that encodes the LHBs protein, and surface promoter II (SPII) is responsible for the 2.1-kb mRNA transcription, which directs the translation of MHBs and SHBs. A previous study comparing the SPI and SPII promoter activities between genotypes A and D revealed a weaker SPII promoter activity in genotype D, whereas the activity of the SPI promoter did not show differences between the two genotypes (Zhang et al., 2017). The weaker SPII promoter activity observed in genotype D might be responsible for the reduced HBsAg production observed in this genotype.

Furthermore, we have observed striking differences in the extra- and intracellular distribution of HBsAg. Genotypes A2 and D1 retained around 50% of the expressed antigen, and genotypes B2 and C1 showed intermediate levels of retention, while nearly all HBsAg was secreted for genotype F1b. As a whole, these results indicate that besides the levels of expression, different factors affect HBsAg secretion. In this regard, the ratio between the different forms of HBsAg is one of the best characterized factors that influence the secretion of HBsAg. It has been shown that HBsAg composition shows specific patterns at different stages of the natural history of chronic infection, as well as high expression levels of LHBs have been associated with intracellular antigen retention (Churin et al., 2015; Pfefferkorn et al., 2018; Lin et al., 2020). This was observed in genotype D1, in which LHBs/MHBs represent 33.6% of the total HBsAg. However, in genotype A2, where almost 50% of the antigen is retained, the amount of LHBs and MHBs represents 13.5% of the total HBsAg. Therefore, other factors might be involved in the intracellular retention of the antigen. In addition, it has been shown that intracellular accumulation of HBsAg triggers a sustained endoplasmic reticulum (ER) overload response, leading to ER oxidative stress, metabolic switching, and genomic instability which are associated with a pro-oncogenic effect (Li et al., 2019; Lin et al., 2020). Therefore, the intracellular accumulation of HBsAg may play a role in inducing liver damage, in particular for genotypes A2 and D1 where half of the expressed antigen is intracellularly accumulated.

Finally, marked differences were also observed in the levels of expression and secretion of HBeAg between the different genotypes. The highest levels were detected for genotype C1, despite its low replication rate, and the lowest for genotypes D1 and F1b. These findings are in line with a recent study by Cooper et al. that showed significantly higher levels of quantitative HBeAg in serum samples of patients chronically infected with genotype C in relation to genotypes B, A1, and D (Cooper et al., 2021). Interestingly, the expression levels of HBeAg do not strictly correlate with those of Precore mRNA, which were higher for genotypes A2 and D1. Hence, other factors besides the Precore mRNA level appear to influence the HbeAg expression and secretion, as might be post-translational modifications of p25 and p22 precursory Precore proteins.

Remarkably, as observed for HbsAg, genotype A2 retained intracellularly about 50% of the expressed HbeAg. It has been described that HbeAg has a dual function, circulating HBeAg (p17) plays an immunomodulatory role during the immunotolerant stage in the natural history of infection, modulating the host’s immune response and contributing to the establishment of persistent viral infection, while cytosolic HBeAg (p22) mainly antagonizes the innate immune responses (Milich and Liang, 2003; Frelin et al., 2009; Milich, 2019). In this regard, previous works have shown that cytosolic HBeAg can suppress IL-1β-mediated NF-κB activation and impair JAK-STAT signaling leading to the repression of interferon action, providing a molecular mechanism that also promotes persistence of infection (Wilson et al., 2011; Mitra et al., 2019). Therefore, the intracellular retention of HBeAg detected in genotype A2 may contribute to counteract the immune pressure, thus favoring viral persistence in up to 10% of the adulthood infection (Kobayashi et al., 2002; Suzuki et al., 2005; Nabuco, 2012).

## Conclusion

This study provides new insights into the biological characteristics of HBV genotypes. The replicative capacity and antigen expression of the most prevalent European, Asian, and American genotypes were comprehensively compared, identifying marked differences in viral replication and protein expression that might contribute to understand the differential role of genotypes in clinical outcomes, progression of liver disease, HBeAg seroconversion rates, and the emergence of mutation profiles.

## Data Availability Statement

The raw data supporting the conclusions of this article will be made available by the authors, without undue reservation.

## Author Contributions

ME performed the experiments, analyzed the data, and drafted the manuscript. LT and LM contributed to the conduction of experiments. ME, JQ, RC, and DF participated in the conception, drafting, and/or editing of the manuscript. All authors have read and agreed to the published version of the manuscript.

## Funding

This work was supported by grants from Consejo Nacional de Investigaciones Científicas y Técnicas (CONICET) [PIP11220080101169], and Universidad de Buenos Aires UBACyT [20020170100206BA 2018-2021], PIDAE [2019-3473].

## Conflict of Interest

The authors declare that the research was conducted in the absence of any commercial or financial relationships that could be construed as a potential conflict of interest.

## Publisher’s Note

All claims expressed in this article are solely those of the authors and do not necessarily represent those of their affiliated organizations, or those of the publisher, the editors and the reviewers. Any product that may be evaluated in this article, or claim that may be made by its manufacturer, is not guaranteed or endorsed by the publisher.

## Footnotes

^1^ https://hbvdb.lyon.inserm.fr/HBVdb/HBVdbIndex

## Supplementary Material

The Supplementary Material for this article can be found at: https://www.frontiersin.org/articles/10.3389/fmicb.2021.758613/full#supplementary-material
