# Supplementary material for: Biological Characterization of Hepatitis B virus Genotypes: Their Role in Viral Replication and Antigen Expression
Source: Front Microbiol. 2021 Nov 4;12:758613. doi: 10.3389/fmicb.2021.758613 (PMC8600256; doi:10.3389/fmicb.2021.758613)
Supplement: Supplementary file 1 [file Data_Sheet_1.docx]

**Supplementary material**

**Supplementary Table 1. Characteristics of patients from whom HBV isolates of different genotypes were recovered.**

| Genotype | Age  (years) | Sex | HBsAg | S/Co | HBeAg | S/Co | Anti-HBe | HBV-DNA (Log_10_ IU/ml) |
| --- | --- | --- | --- | --- | --- | --- | --- | --- |
| A2 | 63 | Male | Positive | 1515 | Positive | > 250 | Negative | 8.2 |
| B2 | 30 | Male | Positive | 945 | Positive | > 250 | Negative | 8.03 |
| C1 | 24 | Male | Positive | 1143 | Positive | > 250 | Negative | 7.8 |
| D1 | 32 | Male | Positive | 1582 | Positive | > 250 | Negative | 7.9 |
| F1b | 24 | Female | Positive | 1224 | Positive | > 250 | Negative | 8.1 |

S/Co: Sample/Cut off value


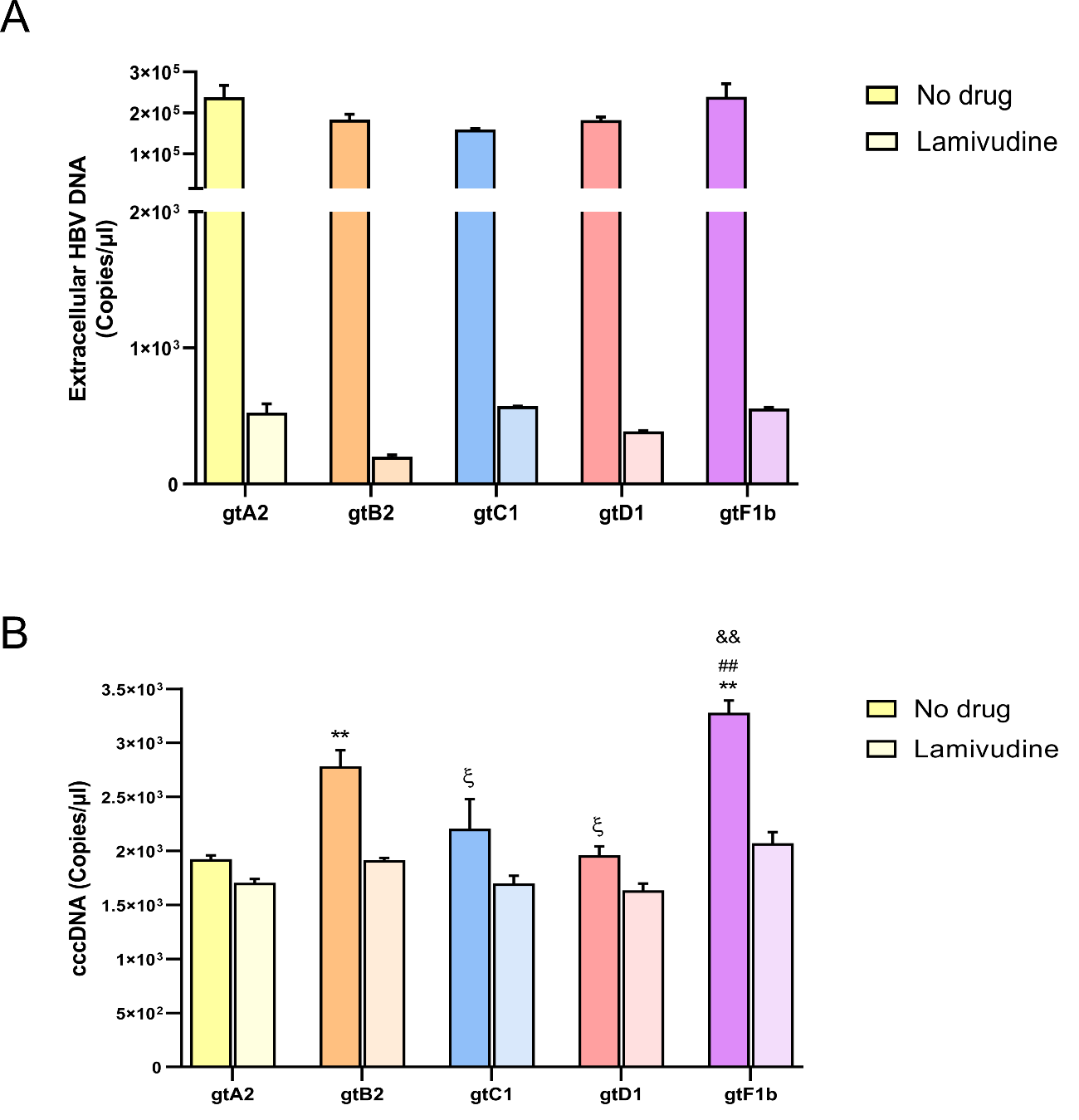


**Supplementary Figure 1.** Effect of Lamivudine on HBV replication among genotypes. HuH-7 cells were transfected with linear full-length HBV genomes of genotypes A2, B2, C1, D1, and F1b in the presence or in the absence of 100 µM Lamivudine. Three days post-transfection, culture supernatants were harvested, and HBV extracellular DNA levels were determined by qPCR. Shown values represent the mean ± standard deviation of three independent experiments.


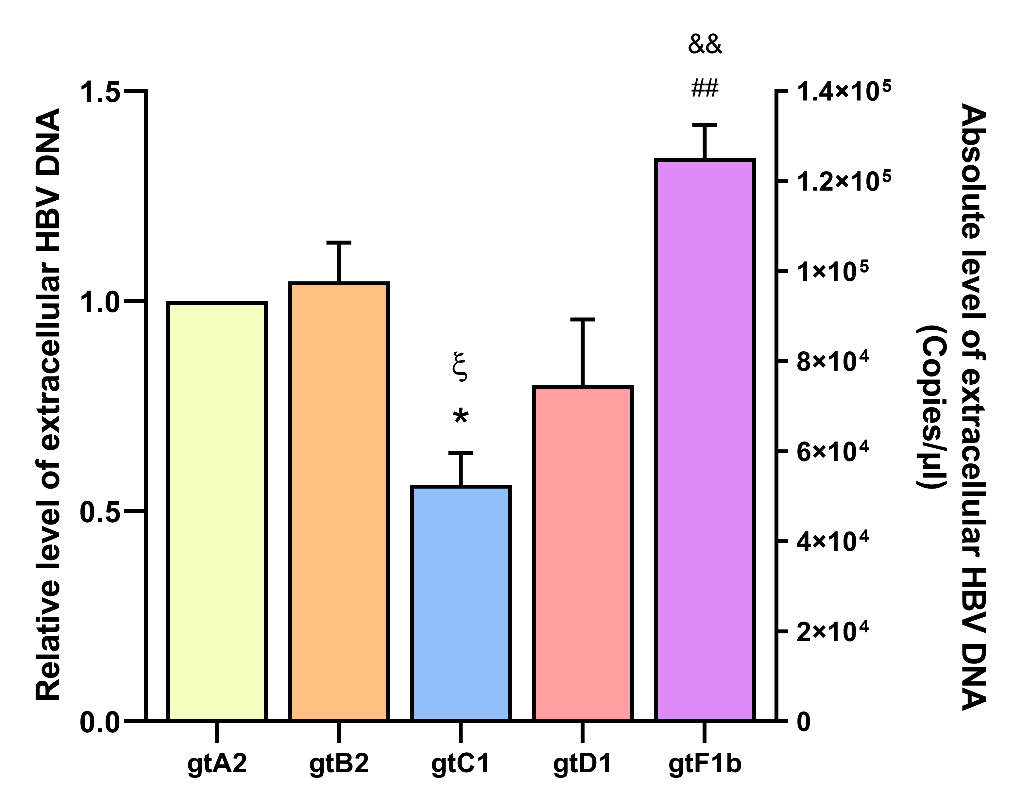


**Supplementary Figure 2.** Analysis of extracellular HBV DNA levels among genotypes. HepG2 cells were transfected with linear full-length HBV genomes of genotypes A2, B2, C1, D1, and F1b. Three days post-transfection culture supernatants were harvested, and extracellular HBV DNA levels were determined by qPCR. Shown values represent the mean ± standard deviation of three independent experiments. *: difference in relation to gtA2, ξ: difference in relation to gtB2, #: difference in relation to gtC1 and &: difference in relation to gtD1. One symbol *p* < 0.005 and two symbols *p* < 0.0001.


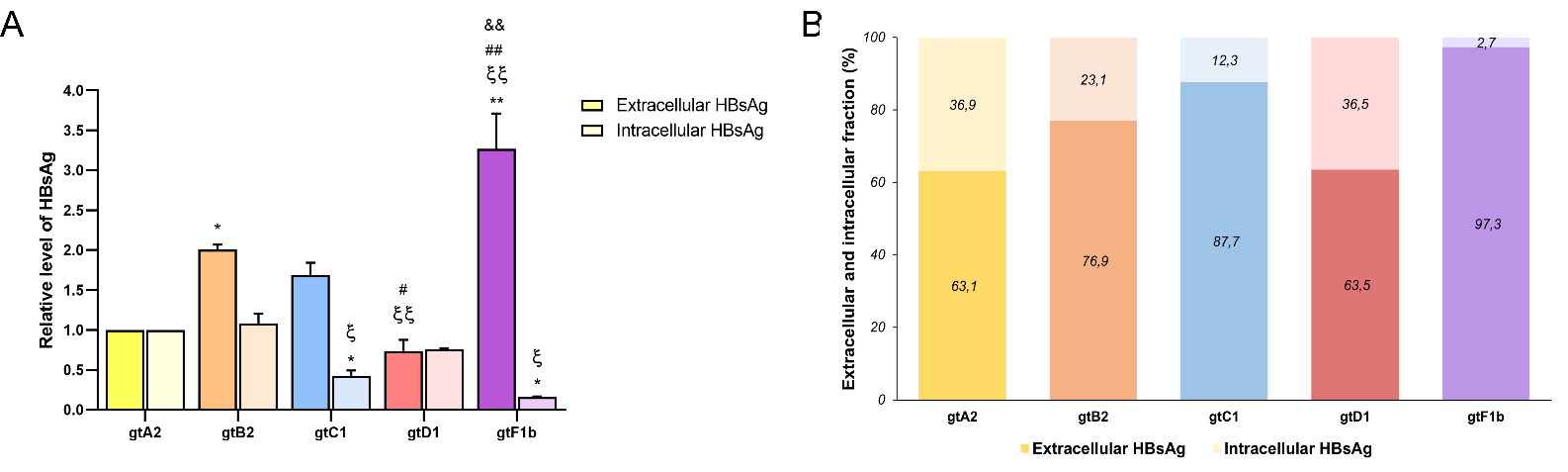


**Supplementary Figure 3.** Analysis of intracellular and secreted HBsAg levels across genotypes. HegG2 cells were transfected with linear full-length HBV genomes of genotypes A2, B2, C1, D1, and F1b. Three days post-transfection, cells and culture supernatants were harvested and intracellular and extracellular levels of HBsAg were determined by electrochemiluminescence immunoassay (A). Extracellular/intracellular HBsAg ratio (B). Values shown represent the mean ± standard deviation of three independent experiments. * Intracellular or extracellular difference in relation to genotype A2, ξ: Intracellular or extracellular difference in relation to genotype B2, #: Intracellular or extracellular difference in relation to genotype gtC1 and &: Intracellular or extracellular difference in relation to genotype D1. One symbol p < 0.005 and two symbols p < 0.0001.


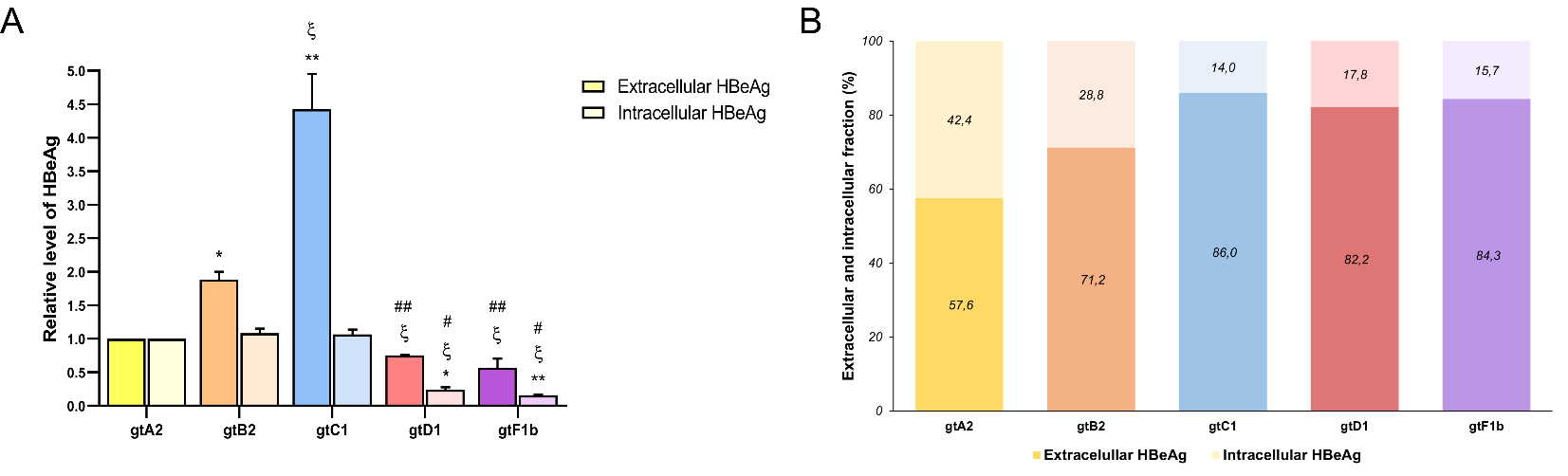


**Supplementary Figure 4.** Analysis of intracellular and secreted HBeAg levels across genotypes. HepG2 cells were transfected with linear full-length HBV genomes of genotypes A2, B2, C1, D1, and F1b. Three days post-transfection, cells and culture supernatants were harvested and intra and extracellular levels of HBeAg were determined by electrochemiluminescence immunoassay (A). Extracellular/intracellular HBeAg ratio (B). Shown values represent the mean ± standard deviation of three independent experiments. * Intracellular or extracellular difference in relation to genotype A2, ξ: Intracellular or extracellular difference in relation to genotype B2, and #: Intracellular or extracellular difference in relation to genotype gtC1. One symbol p < 0.005 and two symbols: *p* < 0.0001.


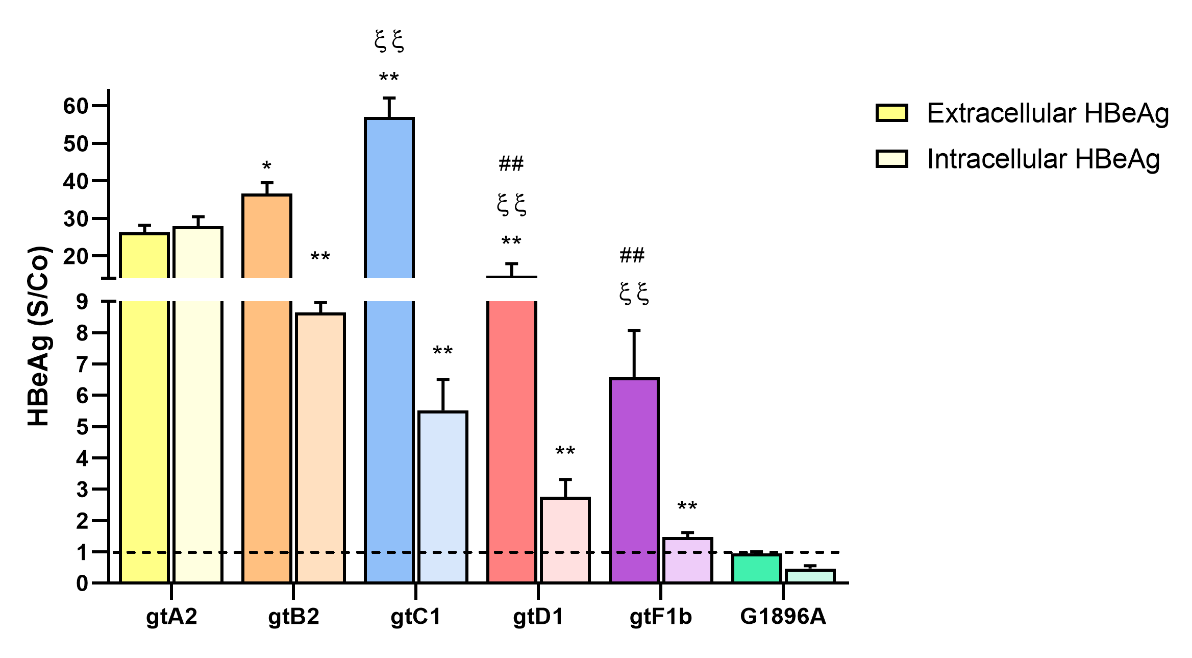


| **Genotype** | **s/Co** | |
| --- | --- | --- |
|  | **Extracellular HBeAg** | **Intracellular HBeAg** |
| A2 | 26.3 ± 1.9 | 27.9 ± 2.6 |
| B2 | 36.7 ± 2.9 | 8.7 ± 0.4 |
| C1 | 57.1 ± 5.0 | 5.5 ± 1.0 |
| D1 | 14.7 ± 3.2 | 2.8 ± 0.6 |
| F1b | 6.6 ± 1.5 | 1.5 ± 0.2 |
| G1896A | 0.9 ± 0.06 | 0.5 ± 0.09 |

**Supplementary Figure 5.** Analysis of intracellular and secreted HBeAg levels across genotypes. HuH-7 cells were transfected with linear full-length HBV genomes of genotypes A2, B2, C1, D1, and F1b, and a full-length HBV genome harboring the G1896A Precore mutation. Three days post-transfection, cells and culture supernatants were harvested and intra and extracellular levels of HBeAg were determined by electrochemiluminescence immunoassay. Results were expressed in Sample/Cut off value (S/CO). Shown values represent the mean ± standard deviation of three independent experiments. *: difference in relation to genotype A2, ξ: difference in relation to genotype B2 and #: difference in relation to gtC1. One symbol p < 0.005 and two symbols: *p* < 0.0001. Dotted line: HBeAg Cut off value.
